# Book Review

**Published:** 1911-03

**Authors:** 


**Serums, Vaccines, and Toxines in Treatment and Diagnosis.** By W. C. Bosanquet and John W. H. Eyre. Pp. viii, 362. Second Edition. London: Cassell and Company Limited. 1910.—The first edition of this exceedingly useful summary of the principles and practice of serum and vaccine therapy was published in 1904, and a second edition is now very opportune. In the former the use of tuberculin and of bacterial vaccines generally claims little space, and the discovery of opsonins is not mentioned. In the second edition these subjects occupy most of the space allotted to new matter. The authors throughout write rather as recorders of work done than as advocates, and maintain a carefully critical attitude. With regard to the use of tuberculin in pulmonary tuberculosis, the conclusion is arrived at that "there seems to be a consensus of opinion that in suitable cases the value of the remedy is beyond question." If so much can be said of perhaps the most difficult and debatable application of vaccine therapy, one is not surprised to find a more emphatic endorsement of success achieved by it in other directions. Whilst subjecting the validity of opsonic index determination to severe criticism, the authors nevertheless are of opinion that it has had at least one important and most beneficial result, in that it has led to a great reduction in both the frequency and the size of the doses of tuberculin generally employed. The effects of minute doses are now recognised, and a great step towards a rational use of the remedy has been taken. Is not this a tacit admission that a useful degree of constancy and accuracy is attainable in this laborious work ? Every worker who has had frequent occasion to chose between alternative diagnoses by aid of opsonic indices, and has afterwards found his choice justified by vaccine treatment, knows that this is so, and the quotation of some instances of discordant determinations on the same serum by different workers is not likely to cause such to set aside a technique which can, on occasion, give invaluable help. As regards serums, anti-bacterial and anti-toxic, the authors confess that, with the exception of anti-diphtheritic serum, results have been on the whole disappointing, and they do not appear convinced of the utility of normal horse serum, though much has been claimed for it. But that our resources against bacterial infections, and our means of diagnosing their obscure constitutional sequelæ, have been greatly increased during the last few years, and are likely to be augmented as investigations of blood plasma become more exhaustive, no impartial reader of this little treatise can doubt.

**Reports of the Sleeping Sickness Commission of the Royal Society.** No. X. London: Darting & Son Ltd. 1910.—Further details in the life-history of the glossina palpalis as a carrier of trypanosoma vivax in Uganda, and some further points on the duration of the infectivity of the glossina after the removal of the lake shore population are here reported. Captain F. P. Mackie, of the Indian Medical Service, is associated with Colonel Sir David Bruce, C.B., F.R.S., in these researches, and his old medical school friends in Bristol will be gratified to see that his time is so well spent.

**Loose-Leaf Medical Pocket Book.** London: John Walker and Co. Ltd. 1910.—The feature of this " visiting list " is the readiness with which the various sections can be renewed, enlarged or altered in any way to suit the individual requirements of any practice, while the use of a transfer case enables the practitioner to keep a permanent record of all cases noted down from day to day. The separate sections, viz. visiting list, addresses, cash account, visits record, accounts rendered, vaccinations, etc., etc., are turned up with the greatest ease. At any rate, it is worth the while of any practitioner to examine this book and judge for himself whether it is suited to his taste and requirements.

**Diseases of the Joints and Spine.** By HOWARD MARSH, M.A., M.C. Cantab. New and Enlarged Edition. Thoroughly Revised by the Author and by C. GORDON WATSON, F.R.C.S. Pp. xv, 632. London: Cassell and Co. Ltd. MCMX.—Many of us have valued very highly Professor Marsh's book on *Diseases of the Joints and Spine*, and we greatly welcome a new and enlarged edition of it. In bringing out this new edition Mr. Gordon Watson has been associated with Professor Marsh.

We notice that it is now fifteen years since the last edition appeared, and there is of course much to add to a work of this kind in that time, and everything seems to have been added which would bring the book thoroughly up to date. We are glad to see that a very definite distinction is now drawn between osteo-arthritis and rheumatoid-arthritis, and doubtless the authors have been influenced in rewriting this chapter by the recent very interesting discussion on this subject at the Royal Society of Medicine. An additional chapter on "Intermittent Hydrarthrosis" has been added. In this condition there is recurrent effusion into one or more joints—very often the knee—and in the intervals the joints seem to be perfectly normal. There may be also severe pain at times in the affected joints. It is a very curious condition, and may be cured by drugs such as quinine or arsenic. A valuable chapter has also been added on Coxa Vara. Professor Marsh has so largely embodied Mr. Elmslie's views on coxa vara in this chapter—it is so largely modelled on his writings—that Professor Marsh might almost have inserted a chapter written by Mr. Elmslie himself, as he has one on hypertrophic pulmonary osteo-arthropathy written by Dr. Waterhouse. We must say we feel unable to conceive of the gradual slipping of the epiphysis in coxa vara. It seems to us more likely that a rapid displacement occurs, but symptoms are not produced until union takes place in malposition.

Professor Marsh has very fully described and illustrated the splint used by Mr. Gauvain at the Cripples' Home at Alton. We do not see that it has any great advantage over the ordinary Phelp's bar, and we feel rather doubtful if the hyperextension of the spine is really desirable. We feel sure that everyone who has known the value of Professor Marsh's earlier edition will be glad to have the present one in addition.

**Difficult Labour.** By G. Ernest Herman, M.B. New and Enlarged Edition. Pp. xii, 547. London: Cassell and Co. Ltd. 1910.—This well-known text-book makes its appearance in its fifth edition as a somewhat thicker volume. It has been revised throughout, additions have been made to the letterpress in numerous places, and a number of new plates inserted. A new chapter on retroversion of the gravid uterus has been added. Although this can hardly be included under "difficult labour," it is a distinctly useful addition. The last chapter on puerperal eclampsia has been included at the request of numerous readers. The author insists on the inexpediency of emptying the uterus as a routine measure. The treatment by morphia gets strong recommendation. The should be read by all in general practice, and the new edition is a distinct improvement on its useful and popular predecessors.

**Practical Obstetrics.** By E. HASTINGS TWEEDY and G. T. WRENCH, M.D. Second Edition. Pp. xx, 491. Oxford Medical Publications. 1910.—A further acquaintance with this work enables us to repeat the good opinion which we expressed concerning it when it first appeared under another title. We think the change of title an improvement, as the book deserves to be recognised as an exposition of midwifery practice generally, and not of the Rotunda methods only, excellent as they are. The book is one that every student and practitioner of obstetrics and gynæcology should have.

**Hints for the General Practitioner in Rhinology and Laryngology.** By Dr. JOHANN FEIN. Translated by J. BOWRING HORGAN, M.B., B.Ch. Pp. xiii, 223. London: Rebman Limited. MCMX.—This work, as its title implies, makes no pretence to be a scientific treatise, but is designed to lend a helping hand to the general practitioner. As such it will be found useful, but there are many defects. The symptomatic classification employed would no doubt have served a purpose had it been found possible to use it throughout; but while under "dysphagia" are grouped the majority of the diseases of the throat, such obvious symptoms as "epistaxis" and "nasal discharge" are not used in the classification of nasal diseases. The subject matter will serve a useful purpose in helping to dispel the all too popular idea that "catarrh" and "nasal polypus" cover the whole of laryngology and rhinology. The importance of hoarseness as an early indication of malignant disease of the larynx is also duly emphasised, as is the importance of early removal of adenoids before they have produced permanent damage. The author appears to attach undue importance to the size of a tonsil, to the neglect of its septicity, as an indication for removal. The sweeping condemnation of enucleation of the tonsil as an alternative to tonsillotomy will not meet with general approval in this country at any rate. Although the removal of adenoids is but briefly described, as being outside the general practitioner's sphere, room is found for a description and illustration of an adenoid curette devised by the author. Diseases of the larynx are dismissed in a very few pages, the use of a laryngeal mirror not being included. It is a pity that such a little practical point as the method of laryngeal auto-insufflation, although mentioned, is not described. Urgent dyspnœa, although usually having to be dealt with by the general practitioner, is not treated in any detail. The illustrations are clear and the translator has done his work well.

**An Introduction to the Study of Hypnotism.** By H. E. WINGFIELD, M.D. Pp. viii, 175. London: Baillèire, Tindall and Cox. 1910.—In a small volume of some 150 pages we have a most lucid and practical description of hypnotism. Though the author disclaims anything new for those who have studied the larger text-books on the subject, the way in which every point is illustrated by an ingenious experiment makes the book refreshingly interesting to read, besides being a distinctly practical work. After a short chapter on the history followed by a simple account of the theory of the subconscious mind, which is the basis of modern hypnotism, the rest of the book is taken up with Phenomena, Treatment, and the Case against Hypnotism. The methods of induction are given concisely, and with a number of useful practical suggestions. The chapter on treatment will probably interest medical men most. There is still much ignorance concerning treatment by hypnotic suggestion in the profession, in spite of the writings of such men as Drs. Lloyd Tuckey and Bramwell, to mention only two. There is still far too much fear of what has been proved to be in experienced medical hands a safe and powerful remedy, a remedy too which is often the only one. There are still too many practitioners who think its use is limited to hysterical conditions. We can cordially recommend this book to the notice of the profession.

**The Suggestive Power of Hypnotism.** By FORBES WINSLOW, M.B. Pp. 90. London: Rebman Ltd. 1910.—This is a booklet written apparently for the general public. It should do good if only by explaining the so-called miracles of Christian Science. But it will also help to dispel some of the fear that the general public still have of hypnotism.

**Annual Report of the Board of Regents of the Smithsonian Institution, 1908.** Washington: Government Printing Office, 1909.—The general appendix of this volume contains many papers of great interest on a variety of subjects, but perhaps in this annual report the papers appeal more to the expert than to the general reader, being as a rule more technical in character than usual. Subjects that are much before the public are treated of, such as military aeronautics, aviation, photo-telegraphy, etc. The paper by Major Squier, U.S.A., on military aeronautics is profusely illustrated by most excellent reproductions of photographs of airships and aeroplanes, and consequently makes quite interesting reading. Medical readers will read with pleasure Ronald Ross's brilliant lecture on "Malaria in Greece," delivered before the Oxford Medical Society. We would also direct attention to Professor Sylvanus Thompson's eloquent appreciation of the "Life and Work of Lord Kelvin," the "Kelvin Lecture" of the Institution of Electrical Engineers.

